# Supplementary material for: Targeting the mSWI/SNF Complex in POU2F-POU2AF Transcription Factor-Driven Malignancies
Source: bioRxiv. 2024 May 24:2024.01.22.576669. Originally published 2024 Jan 25. Preprint. [Version 2] doi: 10.1101/2024.01.22.576669 (PMC10849552; doi:10.1101/2024.01.22.576669)
Supplement: Supplement 3 — Figure S1. Selective essentiality of the mSWI/SNF complex in POU2F3 molecular subtype of SCLC. Related to Figure 1. (A) Fold change of positive and negative controls in CRISPR screen. (B) Beta score for different domains of druggable targeted gene in mSWI/SNF complex in SCLC-P cell lines versus SCLC-A cell lines. RRM, RNA recognition motif. (C) Immunoblot analysis of indicated proteins in SCLC cells post-treatment with varying time points (right) or concentrations (left two experiments, four hours) of AU-15330. Vinculin serves as the control for protein loading in all immunoblots. (D) IC50 value of five days of AU-15330 treatment for different subtypes of SCLC cell lines. (E) Representative dose-response curves of SCLC-P and SCLC-A cells treated with AU-15330 at varying concentration for five days. Figure S2. mSWI/SNF inhibition condenses chromatin at enhancer sites in SCLC cells. Related to Figure 2. (A) Chromatin compaction induced by mSWI/SNF ATPase degradation. Visualization of ATAC-seq read-density in NCI-H1048 (SCLC-P) post-treatment for 4 hours with either vehicle or 1 μM AU-15330. (B) Analysis of fold change and significance level for HOMER motifs that are enriched within sites dependent and independent of the mSWI/SNF complex in NCI-H1048 cells. (C) Genome-wide changes in chromatin accessibility upon AU-15330 treatment for four hours in NCI-H526 (SCLC-P) cells along with genomic annotation of sites that lose physical accessibility (lost) or remain unaltered (retained). (D) Top five de novo motifs (ranked by p-value) of mSWI/SNF-dependent sites (top) and mSWI/SNF-independent sites (bottom) enriched within AU-15330-compacted genomic sites (HOMER, hypergeometric test) in NCI-H526 (SCLC-P) cells. POU2F3 (also known as Oct11) is the top motif. (E) Genome-wide changes in chromatin accessibility upon AU-15330 treatment for 4 hrs in NCI-H1048 (SCLC-P) cells along with genomic annotation of sites that lose physical accessibility (lost) or remain unaltered (retained) [file media-3.pdf]

Figure S1

A

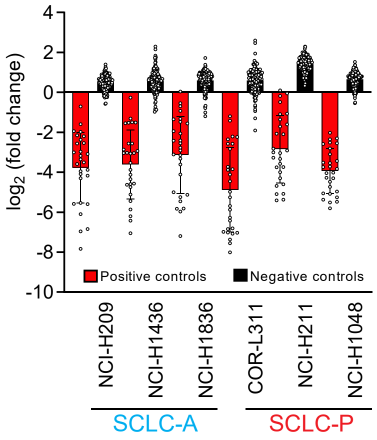

C

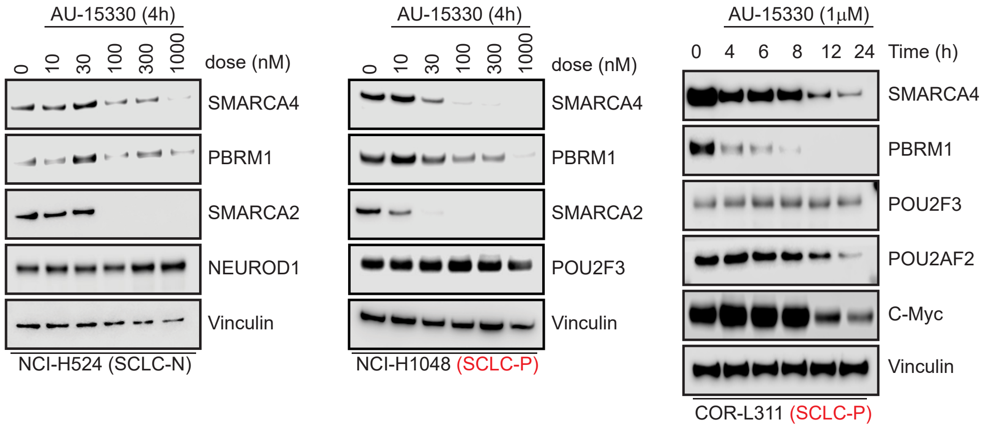

B

sgRNA combinations	sgRNA number	SCLC-P			SCLC-A			Beta score
		COR-L311	NCI-H1048	NCI-H211	NCI-H1436	NCI-H1836	NCI-H209	
sgSMARCA2 (Bromodomain) + sgControl	8	-1.19	0.07	-1.09	-0.03	-0.18	-0.4	-0.54
sgSMARCA4 (Bromodomain) + sgControl	8	-1.83	-0.73	-2.12	-0.2	-0.26	-0.33	-1.29
sgSMARCA2 (Bromodomain) + sgSMARCA4 (Bromodomain)	16	-2.13	-1.00	-2.28	-0.37	-0.7	-0.66	-1.22
sgSMARCA2 (RRM domain) + sgControl	8	-0.39	-0.08	-0.73	0.32	-0.09	-0.05	-0.46
sgSMARCA4 (RRM domain) + sgControl	8	-2.76	-1.39	-2.14	-0.88	-0.66	-0.71	-1.35
sgSMARCA2 (RRM domain) + sgSMARCA4 (RRM domain)	16	-2.14	-1.19	-2.31	-1.05	-0.92	-1.24	-0.81
sgSMARCA2 (Bromodomain) + sgPBRM1 (Bromodomain)	16	-1.62	-0.24	-1.39	-1.11	-0.73	-0.77	-0.21
sgBRD9 (Bromodomain) + sgControl	8	-2.64	-0.49	-1.89	-0.36	-0.51	-1.09	-1.02
sgBRD9 (Bromodomain) + sgBRD7 (Bromodomain)	12	-3.32	-1.15	-2.17	-0.59	-1.03	-1.54	-1.16

D

SCLC cell line	Subtype	AU-15330 (nM)
NCI-H526	SCLC-P	1.8
COR-L311	SCLC-P	8.86
NCI-H1048	SCLC-P	11.45
NCI-H211	SCLC-P	21.91
NCI-H69	SCLC-A	4406
NCI-H1963	SCLC-A	5275
NCI-H209	SCLC-A	8075
COR-L47	SCLC-A	10278
NCI-H1436	SCLC-A	12795
SW1271	SCLC-Y	25460
NCI-H82	SCLC-N	>30000
NCI-H446	SCLC-N	>30000
NCI-H524	SCLC-N	>30000
NCI-H1836	SCLC-A	>30000
HCC-33	SCLC-N	>30000

E

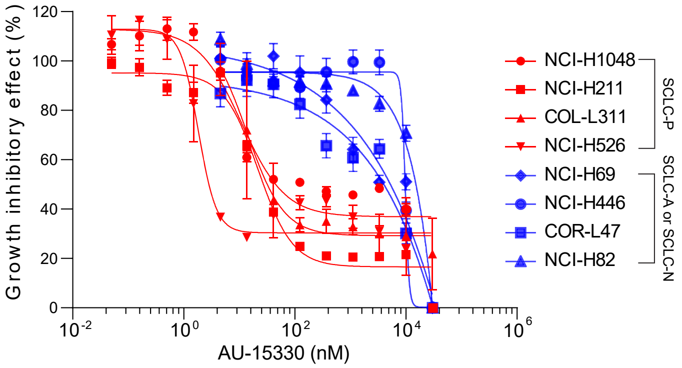

Figure S2

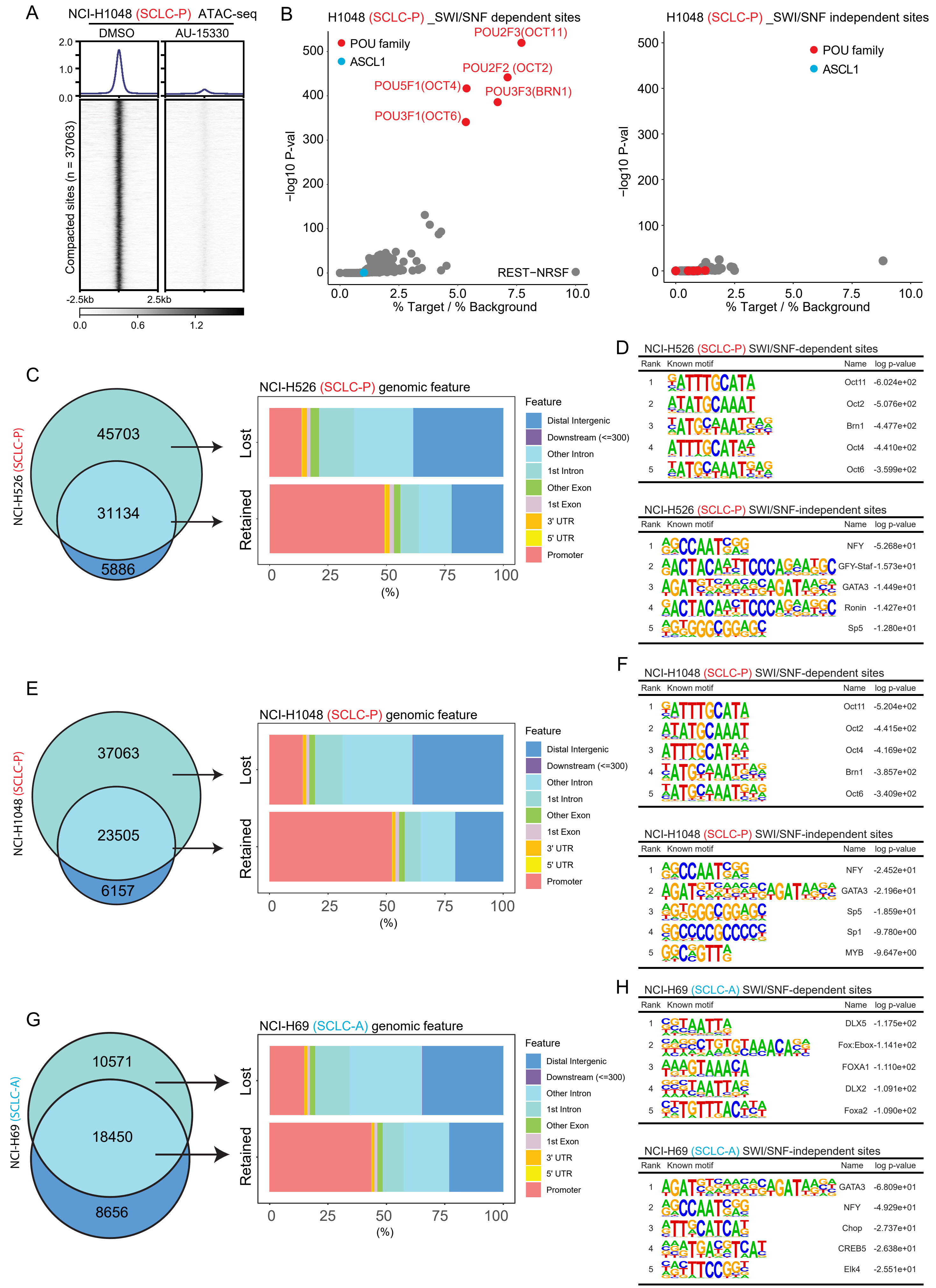

A
NCI-H1048 (SCLC-P) ATAC-seq
DMSO
AU-15330
2.0
1.0
0.0
Compacted sites (n = 37063)
-2.5kb
2.5kb
0.0
0.6
1.2
B
H1048 (SCLC-P) _SWI/SNF dependent sites
POU family
ASCL1
POU2F3(OCT11)
POU2F2 (OCT2)
POU5F1(OCT4)
POU3F3(BRN1)
POU3F1(OCT6)
REST−NRSF
−log10 P-val
% Target / % Background
H1048 (SCLC-P) _SWI/SNF independent sites
C
NCI-H526 (SCLC-P)
45703
31134
5886
NCI-H526 (SCLC-P) genomic feature
Lost
Retained
(%)
Feature
Distal Intergenic
Downstream (<=300)
Other Intron
1st Intron
Other Exon
1st Exon
3' UTR
5' UTR
Promoter
D
NCI-H526 (SCLC-P) SWI/SNF-dependent sites
Rank Known motif Name log p-value
1 Oct11 -6.024e+02
2 Oct2 -5.076e+02
3 Brn1 -4.477e+02
4 Oct4 -4.410e+02
5 Oct6 -3.599e+02
NCI-H526 (SCLC-P) SWI/SNF-independent sites
Rank Known motif Name log p-value
1 NFY -5.268e+01
2 GFY-Staf -1.573e+01
3 GATA3 -1.449e+01
4 Ronin -1.427e+01
5 Sp5 -1.280e+01
E
NCI-H1048 (SCLC-P)
37063
23505
6157
NCI-H1048 (SCLC-P) genomic feature
F
NCI-H1048 (SCLC-P) SWI/SNF-dependent sites
Rank Known motif Name log p-value
1 Oct11 -5.204e+02
2 Oct2 -4.415e+02
3 Oct4 -4.169e+02
4 Brn1 -3.857e+02
5 Oct6 -3.409e+02
NCI-H1048 (SCLC-P) SWI/SNF-independent sites
Rank Known motif Name log p-value
1 NFY -2.452e+01
2 GATA3 -2.196e+01
3 Sp5 -1.859e+01
4 Sp1 -9.780e+00
5 MYB -9.647e+00
G
NCI-H69 (SCLC-A)
10571
18450
8656
NCI-H69 (SCLC-A) genomic feature
H
NCI-H69 (SCLC-A) SWI/SNF-dependent sites
Rank Known motif Name log p-value
1 DLX5 -1.175e+02
2 Fox:Ebox -1.141e+02
3 FOXA1 -1.110e+02
4 DLX2 -1.091e+02
5 Foxa2 -1.090e+02
NCI-H69 (SCLC-A) SWI/SNF-independent sites
Rank Known motif Name log p-value
1 GATA3 -6.809e+01
2 NFY -4.929e+01
3 Chop -2.737e+01
4 CREB5 -2.638e+01
5 Elk4 -2.551e+01

Figure S3

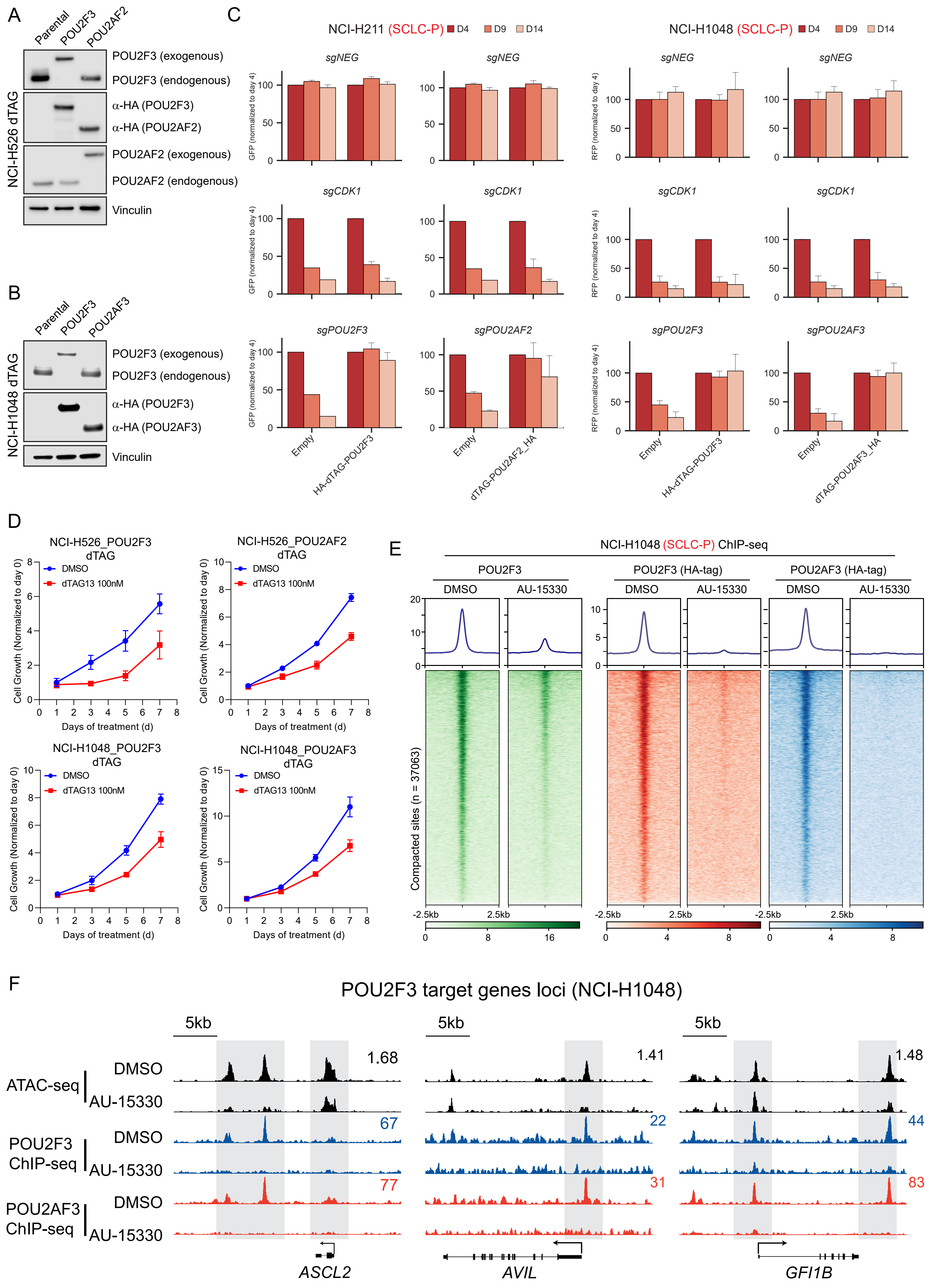
A
Parental
POU2F3
POU2AF2
NCI-H526 dTAG
POU2F3 (exogenous)
POU2F3 (endogenous)
α-HA (POU2F3)
α-HA (POU2AF2)
POU2AF2 (exogenous)
POU2AF2 (endogenous)
Vinculin
B
Parental
POU2F3
POU2AF3
NCI-H1048 dTAG
POU2F3 (exogenous)
POU2F3 (endogenous)
α-HA (POU2F3)
α-HA (POU2AF3)
Vinculin
C
NCI-H211 (SCLC-P) D4 D9 D14
NCI-H1048 (SCLC-P) D4 D9 D14
sgNEG
sgCDK1
sgPOU2F3
sgPOU2AF2
sgPOU2AF3
GFP (normalized to day 4)
RFP (normalized to day 4)
Empty
HA-dTAG-POU2F3
dTAG-POU2AF2_HA
dTAG-POU2AF3_HA
D
NCI-H526_POU2F3 dTAG
NCI-H526_POU2AF2 dTAG
NCI-H1048_POU2F3 dTAG
NCI-H1048_POU2AF3 dTAG
DMSO
dTAG13 100nM
Cell Growth (Normalized to day 0)
Days of treatment (d)
E
NCI-H1048 (SCLC-P) ChIP-seq
POU2F3
POU2F3 (HA-tag)
POU2AF3 (HA-tag)
DMSO
AU-15330
Compacted sites (n = 37063)
-2.5kb
2.5kb
F
POU2F3 target genes loci (NCI-H1048)
5kb
ATAC-seq
POU2F3 ChIP-seq
POU2AF3 ChIP-seq
DMSO
AU-15330
1.68
67
77
1.41
22
31
1.48
44
83
ASCL2
AVIL
GFI1B

Figure S4

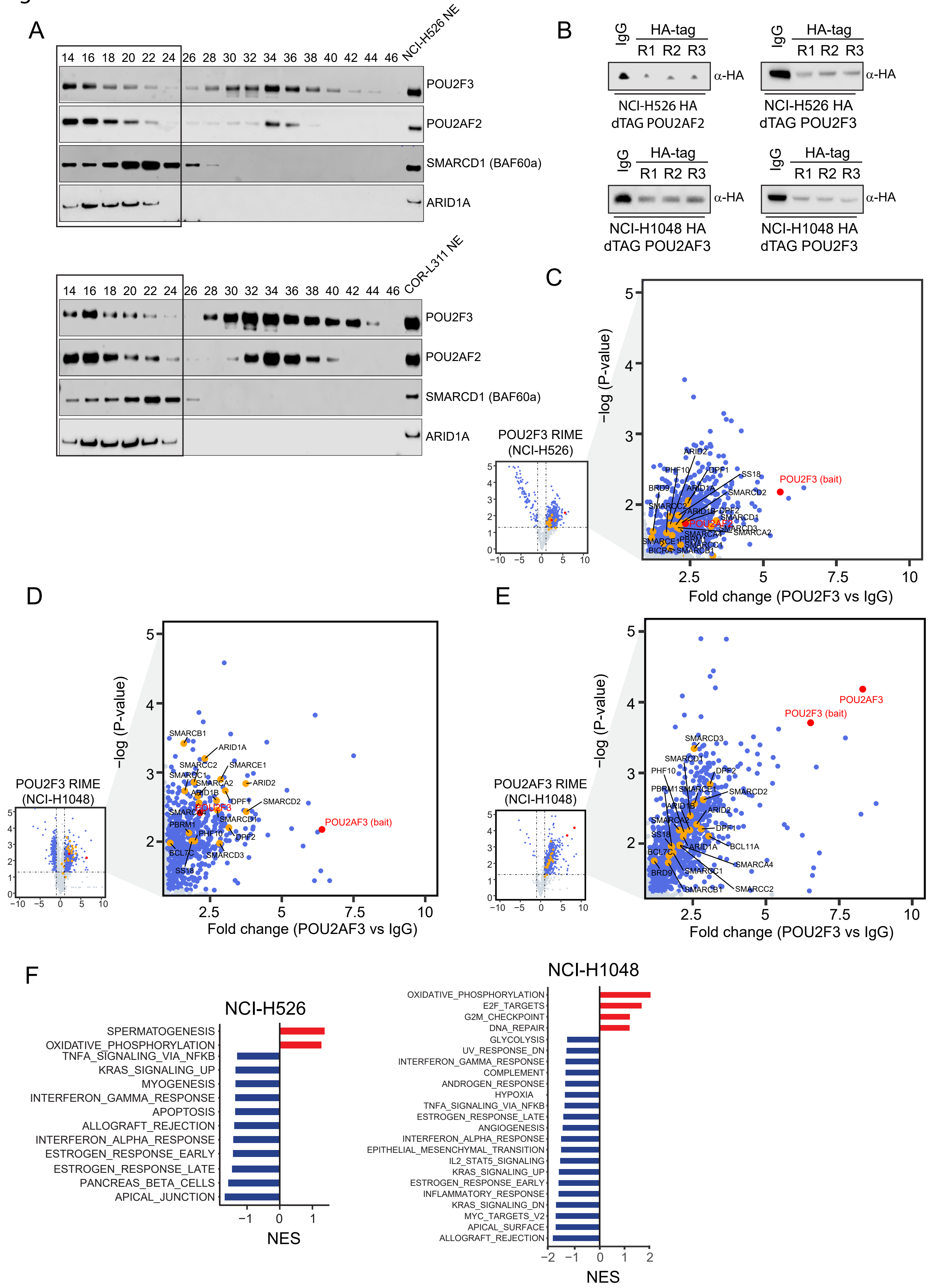
A
14 16 18 20 22 24 26 28 30 32 34 36 38 40 42 44 46
NCI-H526 NE
POU2F3
POU2AF2
SMARCD1 (BAF60a)
ARID1A
COR-L311 NE
B
IgG
HA-tag
R1 R2 R3
α-HA
NCI-H526 HA dTAG POU2AF2
NCI-H526 HA dTAG POU2F3
NCI-H1048 HA dTAG POU2AF3
NCI-H1048 HA dTAG POU2F3
C
POU2F3 RIME (NCI-H526)
−log (P-value)
Fold change (POU2F3 vs IgG)
POU2F3 (bait)
D
POU2F3 RIME (NCI-H1048)
Fold change (POU2AF3 vs IgG)
POU2AF3 (bait)
E
POU2AF3 RIME (NCI-H1048)
Fold change (POU2F3 vs IgG)
POU2AF3
POU2F3 (bait)
F
NCI-H526
SPERMATOGENESIS
OXIDATIVE_PHOSPHORYLATION
TNFA_SIGNALING_VIA_NFKB
KRAS_SIGNALING_UP
MYOGENESIS
INTERFERON_GAMMA_RESPONSE
APOPTOSIS
ALLOGRAFT_REJECTION
INTERFERON_ALPHA_RESPONSE
ESTROGEN_RESPONSE_EARLY
ESTROGEN_RESPONSE_LATE
PANCREAS_BETA_CELLS
APICAL_JUNCTION
NES
NCI-H1048
OXIDATIVE_PHOSPHORYLATION
E2F_TARGETS
G2M_CHECKPOINT
DNA_REPAIR
GLYCOLYSIS
UV_RESPONSE_DN
INTERFERON_GAMMA_RESPONSE
COMPLEMENT
ANDROGEN_RESPONSE
HYPOXIA
TNFA_SIGNALING_VIA_NFKB
ESTROGEN_RESPONSE_LATE
INTERFERON_ALPHA_RESPONSE
ANGIOGENESIS
EPITHELIAL_MESENCHYMAL_TRANSITION
IL2_STAT5_SIGNALING
KRAS_SIGNALING_UP
ESTROGEN_RESPONSE_EARLY
INFLAMMATORY_RESPONSE
KRAS_SIGNALING_DN
MYC_TARGETS_V2
APICAL_SURFACE
ALLOGRAFT_REJECTION
NES

Figure S5

A

SCLC cell line	Subtype	AU-24118 (nM)
NCI-H526	SCLC-P	3.01
NCI-H1048	SCLC-P	7.16
NCI-H211	SCLC-P	5.48
COR-L311	SCLC-P	20.59
NCI-H1436	SCLC-A	2210
COR-L47	SCLC-A	2442
NCI-H1963	SCLC-A	2599
NCI-H1836	SCLC-A	6588
NCI-H209	SCLC-A	8060
NCI-H446	SCLC-N	8605
HCC-33	SCLC-N	8905
NCI-H69	SCLC-A	10052
NCI-H524	SCLC-N	12015
NCI-H82	SCLC-N	13169
SW1271	SCLC-Y	>30000

B

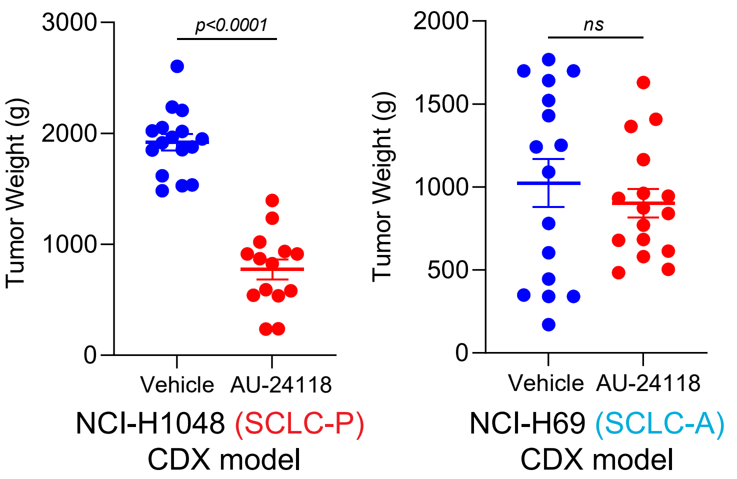

C

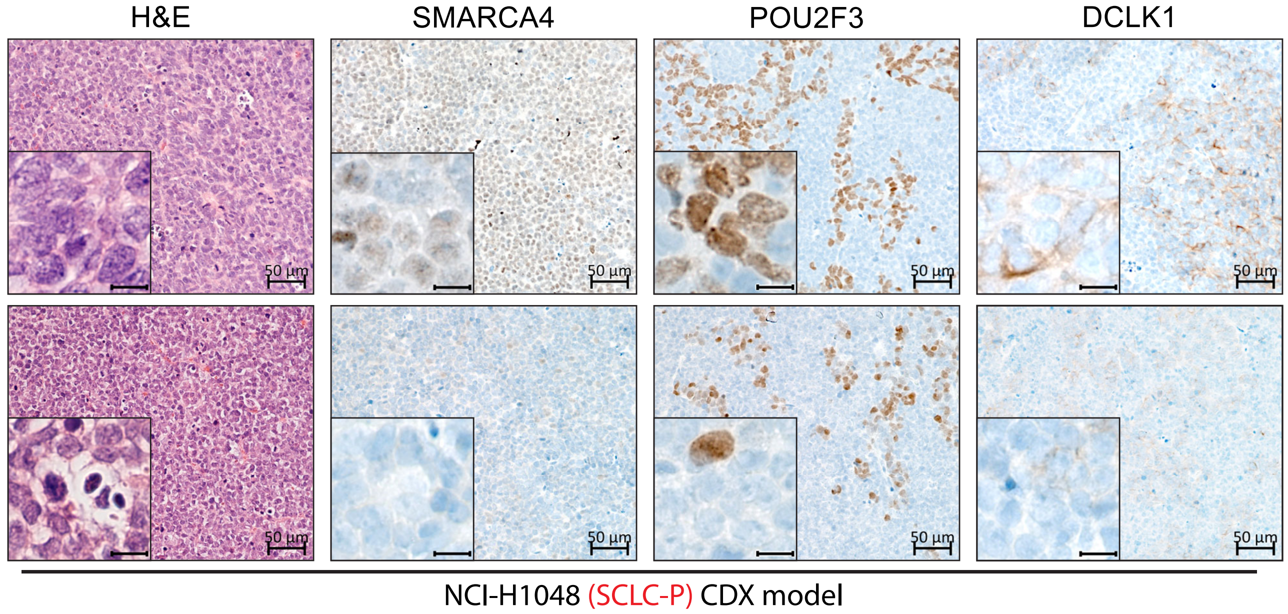

D

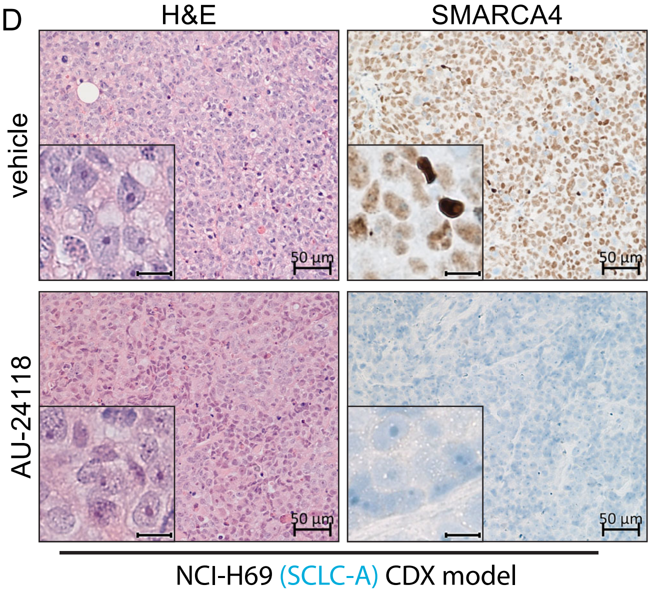

Figure S6

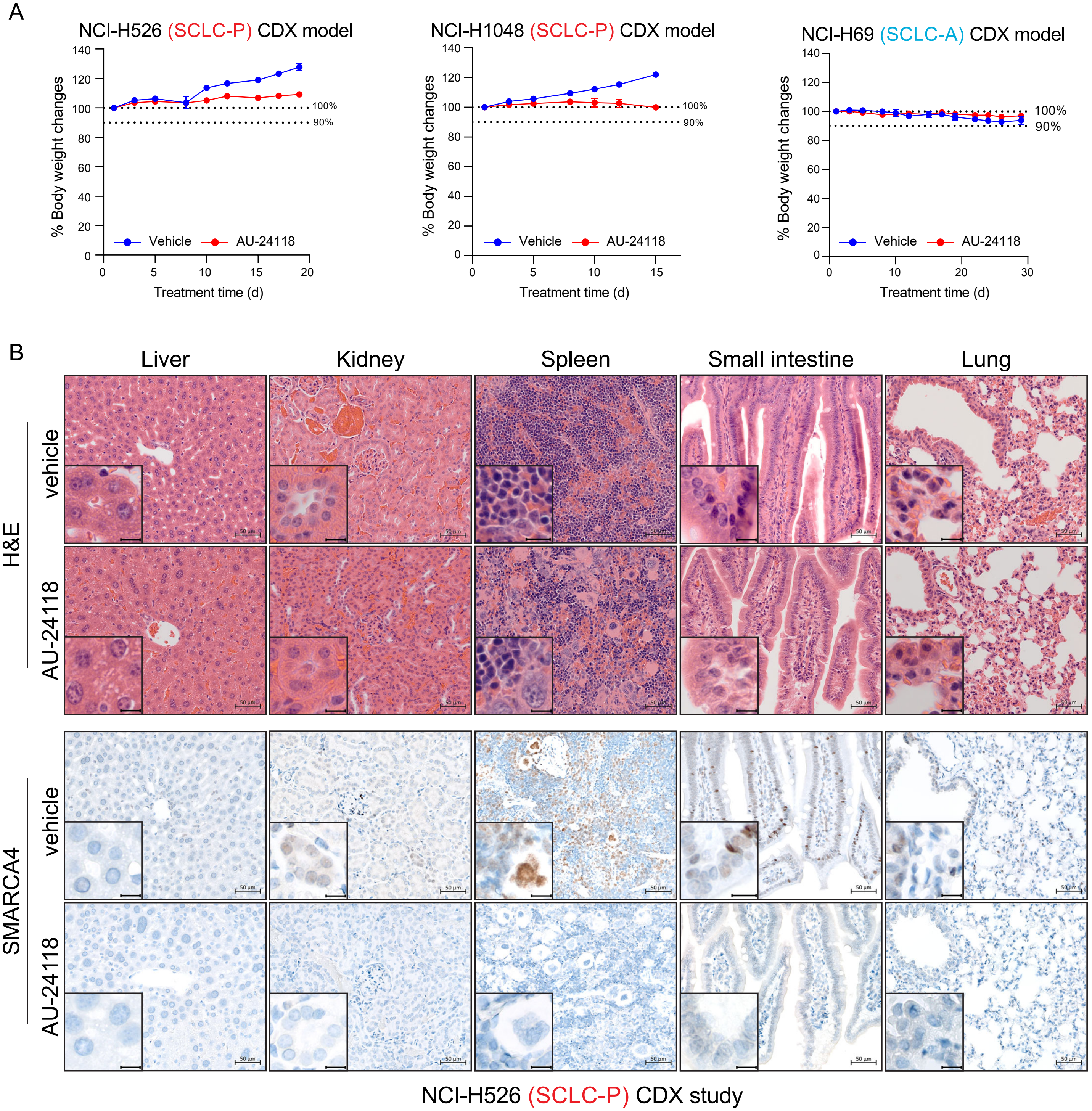

A
NCI-H526 (SCLC-P) CDX model
NCI-H1048 (SCLC-P) CDX model
NCI-H69 (SCLC-A) CDX model
% Body weight changes
Treatment time (d)
Vehicle
AU-24118
100%
90%
B
Liver
Kidney
Spleen
Small intestine
Lung
H&E
SMARCA4
vehicle
AU-24118
NCI-H526 (SCLC-P) CDX study

Figure S7

A

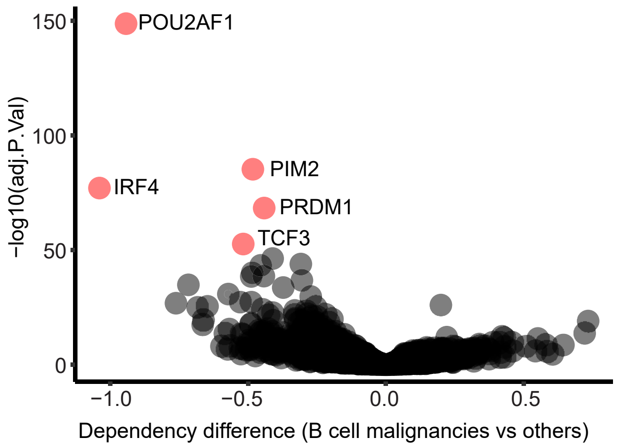

B

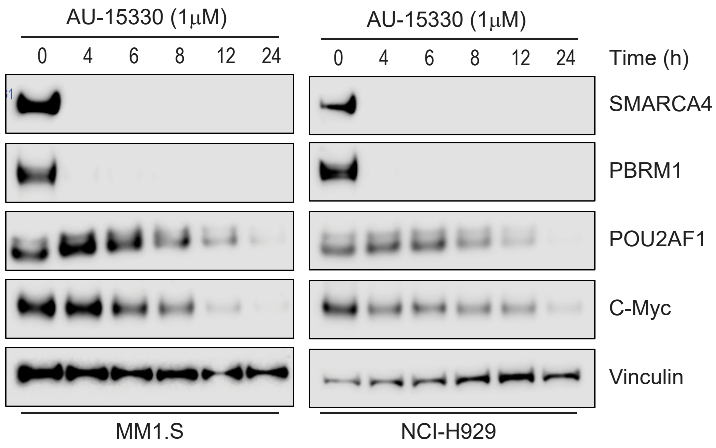

C

MM cell line	AU-15330 (nM)
Karpas-25	2.5
NCI-H929	5.02
MM1.S	9.74
JIM1	20.76
JIM3	32.16
ANBL6	138.8
Karpas-929	145.7
Karpas-417	239.8
RPMI-8226	470.6
U266B1	1542

IC_{50} summary of AU-15330 in MM cell lines

D

DLBCL cell line	Subtype	AU-15330 (nM)
Karpas-422	GCB	22.83
HT	GCB	32.8
Pfeiffer	GCB	34.37
SU-DHL-6	GCB	58.87
DoHH2	GCB	73.45
OCI-Ly1	GCB	164.4
OCI-Ly7	GCB	172.5
SU-DHL-5	GCB	306.5
WSU-DLCL2	GCB	336.5
DB	GCB	363.6
SU-DHL-4	ABC	370.1
TMD8	ABC	370.9
VAL	ABC	439.1
OCI-Ly3	ABC	496.9
U2932	ABC	553.2
RC-K8	GCB or ABC	>30000

IC_{50} summary of AU-15330 in DLBCL cell lines

Figure S8

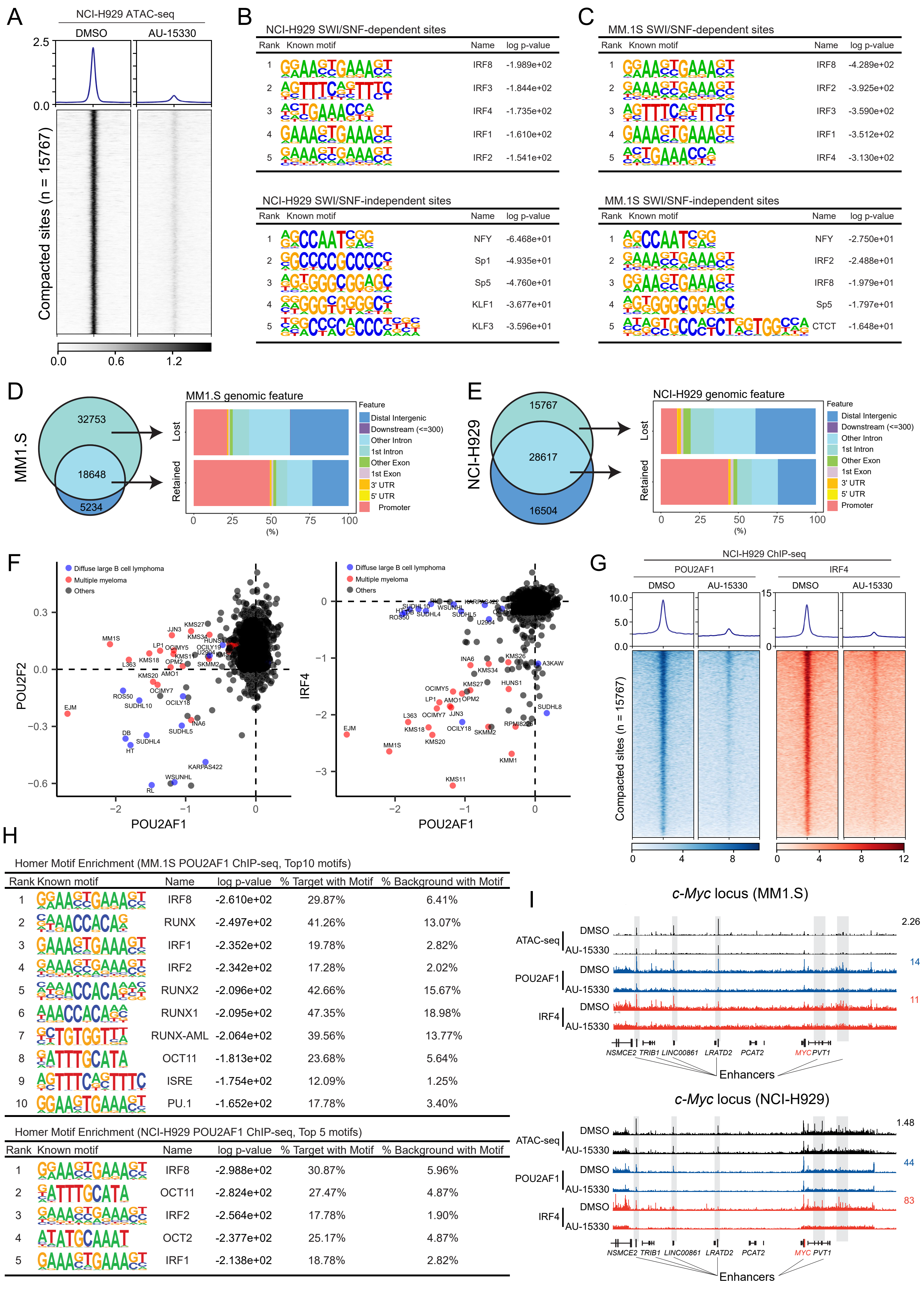
A
NCI-H929 ATAC-seq
DMSO
AU-15330
Compacted sites (n = 15767)
B
NCI-H929 SWI/SNF-dependent sites
Rank Known motif Name log p-value
1 IRF8 -1.989e+02
2 IRF3 -1.844e+02
3 IRF4 -1.735e+02
4 IRF1 -1.610e+02
5 IRF2 -1.541e+02
NCI-H929 SWI/SNF-independent sites
Rank Known motif Name log p-value
1 NFY -6.468e+01
2 Sp1 -4.935e+01
3 Sp5 -4.760e+01
4 KLF1 -3.677e+01
5 KLF3 -3.596e+01
C
MM.1S SWI/SNF-dependent sites
Rank Known motif Name log p-value
1 IRF8 -4.289e+02
2 IRF2 -3.925e+02
3 IRF3 -3.590e+02
4 IRF1 -3.512e+02
5 IRF4 -3.130e+02
MM.1S SWI/SNF-independent sites
Rank Known motif Name log p-value
1 NFY -2.750e+01
2 IRF2 -2.488e+01
3 IRF8 -1.979e+01
4 Sp5 -1.797e+01
5 CTCT -1.648e+01
D
MM1.S
32753
18648
5234
MM1.S genomic feature
Lost
Retained
(%)
Feature
Distal Intergenic
Downstream (<=300)
Other Intron
1st Intron
Other Exon
1st Exon
3' UTR
5' UTR
Promoter
E
NCI-H929
15767
28617
16504
NCI-H929 genomic feature
Lost
Retained
(%)
F
Diffuse large B cell lymphoma
Multiple myeloma
Others
POU2F2
POU2AF1
IRF4
G
NCI-H929 ChIP-seq
POU2AF1
IRF4
DMSO
AU-15330
Compacted sites (n = 15767)
H
Homer Motif Enrichment (MM.1S POU2AF1 ChIP-seq, Top10 motifs)
Rank Known motif Name log p-value % Target with Motif % Background with Motif
1 IRF8 -2.610e+02 29.87% 6.41%
2 RUNX -2.497e+02 41.26% 13.07%
3 IRF1 -2.352e+02 19.78% 2.82%
4 IRF2 -2.342e+02 17.28% 2.02%
5 RUNX2 -2.096e+02 42.66% 15.67%
6 RUNX1 -2.095e+02 47.35% 18.98%
7 RUNX-AML -2.064e+02 39.56% 13.77%
8 OCT11 -1.813e+02 23.68% 5.64%
9 ISRE -1.754e+02 12.09% 1.25%
10 PU.1 -1.652e+02 17.78% 3.40%
Homer Motif Enrichment (NCI-H929 POU2AF1 ChIP-seq, Top 5 motifs)
Rank Known motif Name log p-value % Target with Motif % Background with Motif
1 IRF8 -2.988e+02 30.87% 5.96%
2 OCT11 -2.824e+02 27.47% 4.87%
3 IRF2 -2.564e+02 17.78% 1.90%
4 OCT2 -2.377e+02 25.17% 4.87%
5 IRF1 -2.138e+02 18.78% 2.82%
I
c-Myc locus (MM1.S)
ATAC-seq
POU2AF1
IRF4
DMSO
AU-15330
2.26
14
11
NSMCE2 TRIB1 LINC00861 LRATD2 PCAT2 MYC PVT1
Enhancers
c-Myc locus (NCI-H929)
1.48
44
83
Enhancers

Figure S9

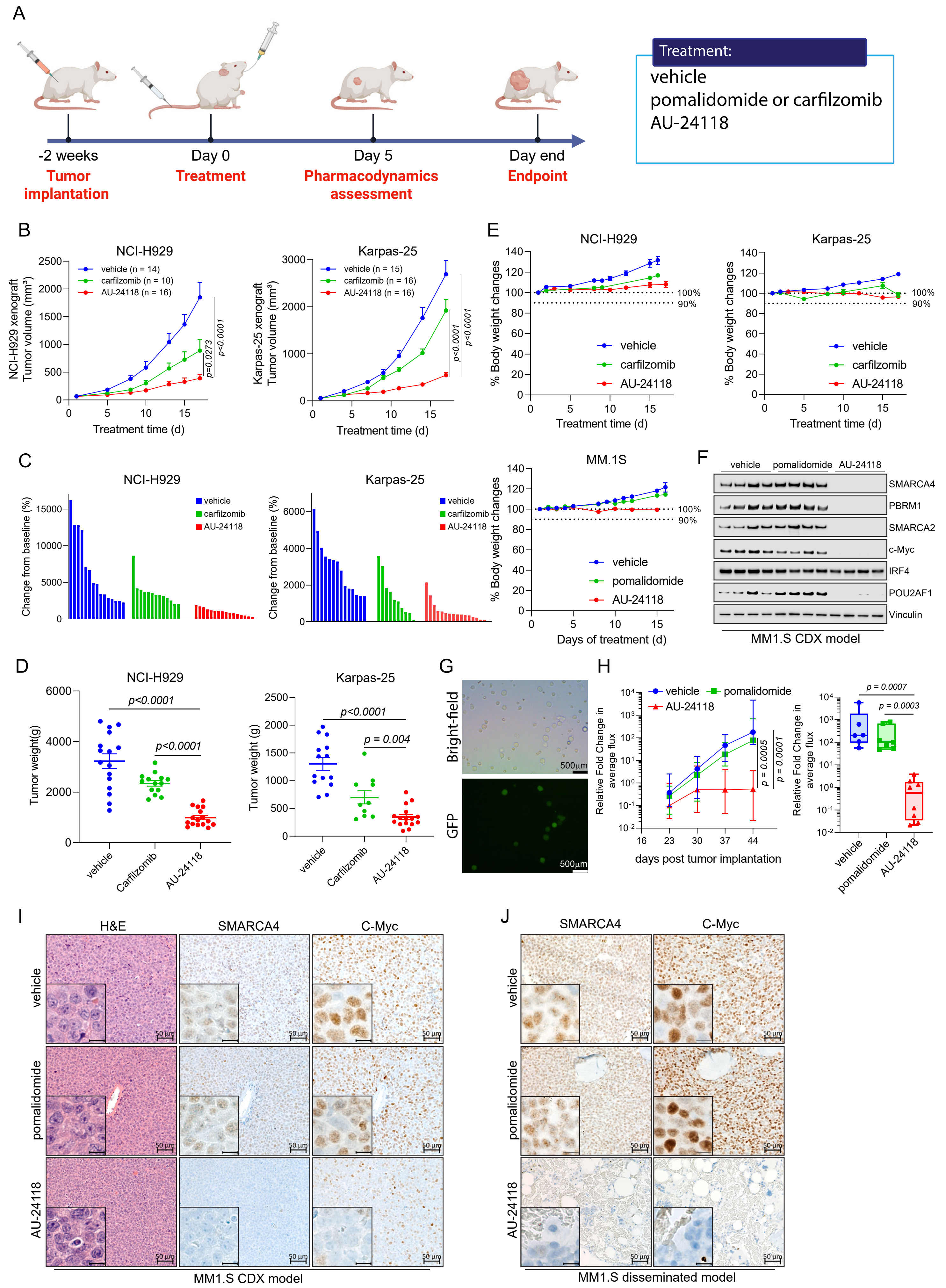
A
-2 weeks
Tumor implantation
Day 0
Treatment
Day 5
Pharmacodynamics assessment
Day end
Endpoint
Treatment:
vehicle
pomalidomide or carfilzomib
AU-24118
B
NCI-H929
vehicle (n = 14)
carfilzomib (n = 10)
AU-24118 (n = 16)
NCI-H929 xenograft Tumor volume (mm³)
Treatment time (d)
p=0.0273
p<0.0001
Karpas-25
vehicle (n = 15)
carfilzomib (n = 16)
AU-24118 (n = 16)
Karpas-25 xenograft Tumor volume (mm³)
p<0.0001
p<0.0001
E
NCI-H929
Karpas-25
% Body weight changes
100%
90%
vehicle
carfilzomib
AU-24118
C
NCI-H929
Karpas-25
Change from baseline (%)
MM.1S
pomalidomide
Days of treatment (d)
F
vehicle
pomalidomide
AU-24118
SMARCA4
PBRM1
SMARCA2
c-Myc
IRF4
POU2AF1
Vinculin
MM1.S CDX model
D
NCI-H929
Tumor weight(g)
p<0.0001
p<0.0001
Karpas-25
Tumor weight (g)
p<0.0001
p = 0.004
vehicle
Carfilzomib
AU-24118
G
Bright-field
GFP
500μm
H
Relative Fold Change in average flux
days post tumor implantation
p = 0.0005
p = 0.0001
p = 0.0007
p = 0.0003
I
H&E
SMARCA4
C-Myc
MM1.S CDX model
J
MM1.S disseminated model
50 μm

Figure 1E

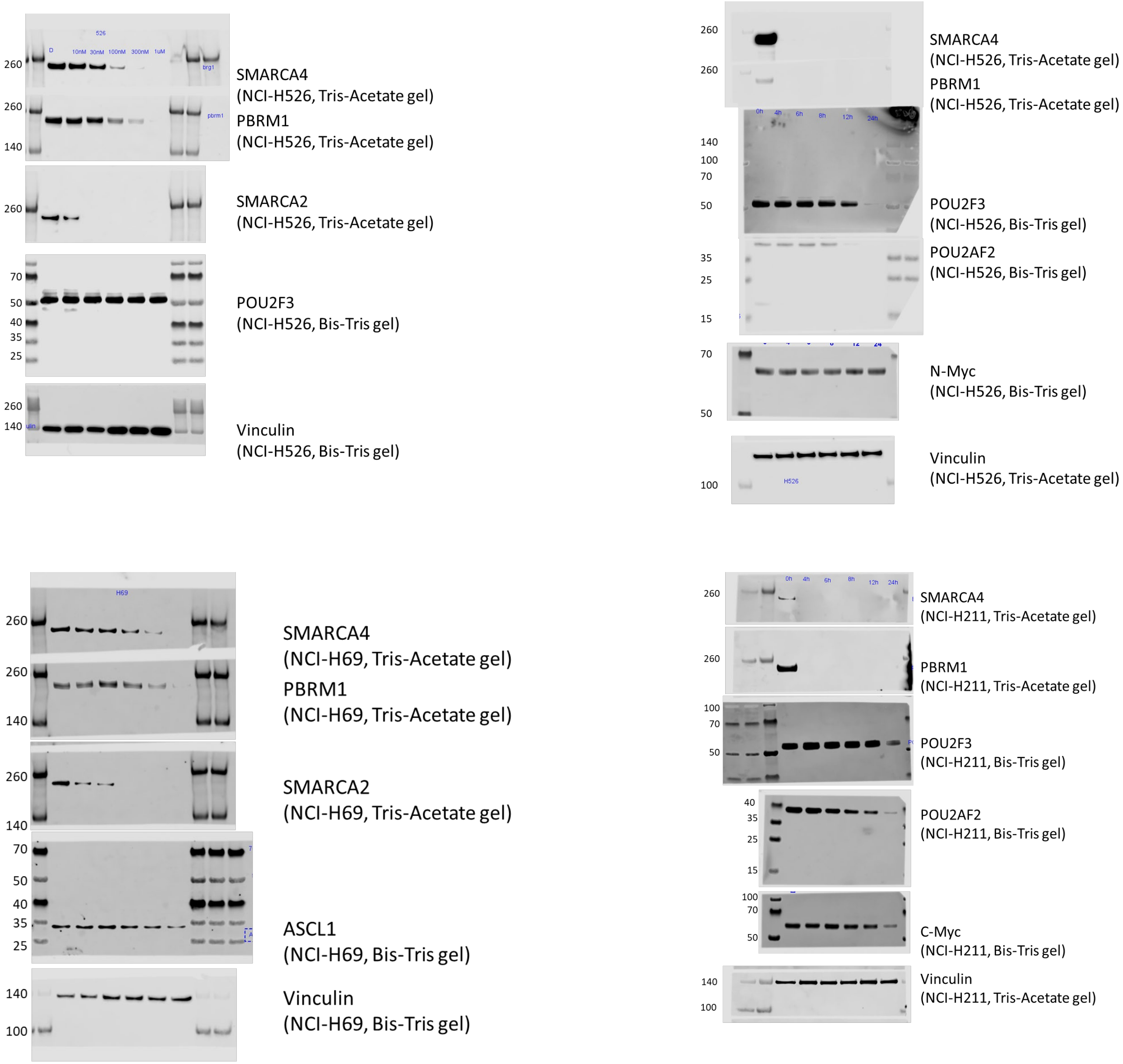
SMARCA4
(NCI-H526, Tris-Acetate gel)
PBRM1
(NCI-H526, Tris-Acetate gel)
SMARCA2
(NCI-H526, Tris-Acetate gel)
POU2F3
(NCI-H526, Bis-Tris gel)
Vinculin
(NCI-H526, Bis-Tris gel)
SMARCA4
(NCI-H526, Tris-Acetate gel)
PBRM1
(NCI-H526, Tris-Acetate gel)
POU2F3
(NCI-H526, Bis-Tris gel)
POU2AF2
(NCI-H526, Bis-Tris gel)
N-Myc
(NCI-H526, Bis-Tris gel)
Vinculin
(NCI-H526, Tris-Acetate gel)
SMARCA4
(NCI-H69, Tris-Acetate gel)
PBRM1
(NCI-H69, Tris-Acetate gel)
SMARCA2
(NCI-H69, Tris-Acetate gel)
ASCL1
(NCI-H69, Bis-Tris gel)
Vinculin
(NCI-H69, Bis-Tris gel)
SMARCA4
(NCI-H211, Tris-Acetate gel)
PBRM1
(NCI-H211, Tris-Acetate gel)
POU2F3
(NCI-H211, Bis-Tris gel)
POU2AF2
(NCI-H211, Bis-Tris gel)
C-Myc
(NCI-H211, Bis-Tris gel)
Vinculin
(NCI-H211, Tris-Acetate gel)

Fig S1C

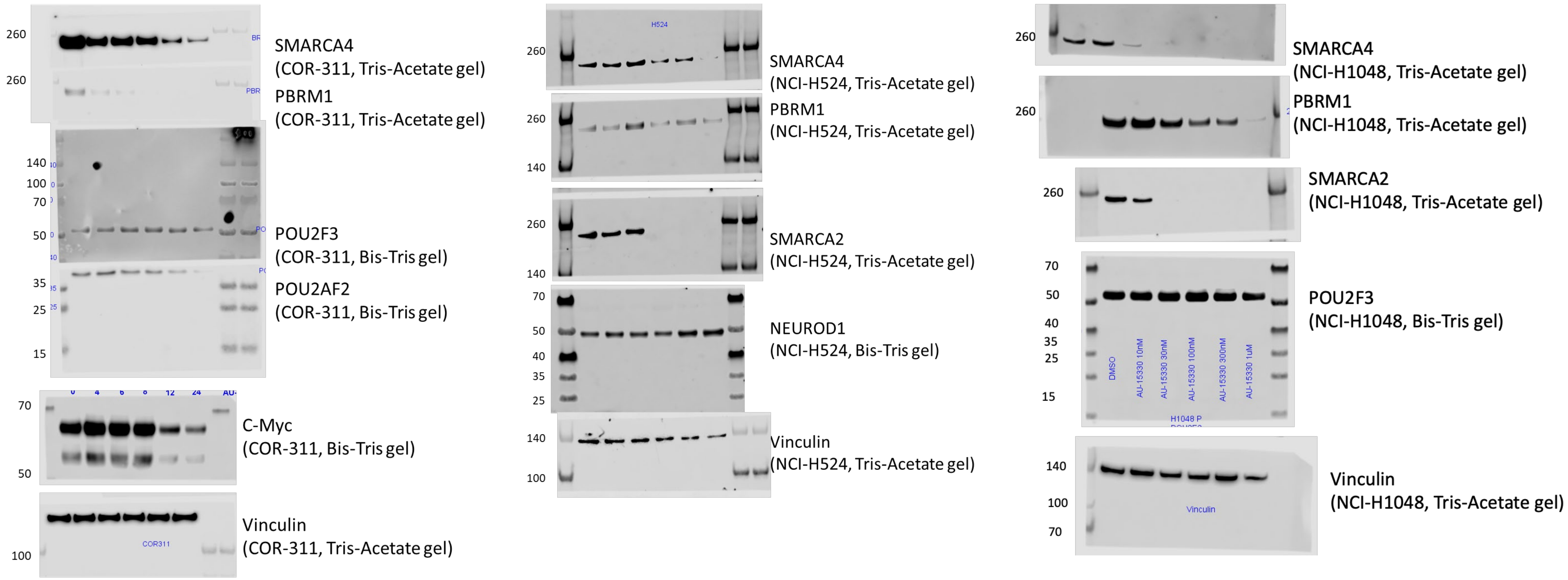
260
SMARCA4
(COR-311, Tris-Acetate gel)
260
PBRM1
(COR-311, Tris-Acetate gel)
140
100
70
50
POU2F3
(COR-311, Bis-Tris gel)
35
25
15
POU2AF2
(COR-311, Bis-Tris gel)
0
4
6
8
12
24
70
50
C-Myc
(COR-311, Bis-Tris gel)
COR311
100
Vinculin
(COR-311, Tris-Acetate gel)
H524
260
SMARCA4
(NCI-H524, Tris-Acetate gel)
260
140
PBRM1
(NCI-H524, Tris-Acetate gel)
260
140
SMARCA2
(NCI-H524, Tris-Acetate gel)
70
50
40
35
25
NEUROD1
(NCI-H524, Bis-Tris gel)
140
100
Vinculin
(NCI-H524, Tris-Acetate gel)
260
SMARCA4
(NCI-H1048, Tris-Acetate gel)
260
PBRM1
(NCI-H1048, Tris-Acetate gel)
260
SMARCA2
(NCI-H1048, Tris-Acetate gel)
70
50
40
35
25
15
DMSO
AU-15330 10nM
AU-15330 30nM
AU-15330 100nM
AU-15330 300nM
AU-15330 1uM
H1048 P
POU2F3
(NCI-H1048, Bis-Tris gel)
140
100
70
Vinculin
Vinculin
(NCI-H1048, Tris-Acetate gel)

Fig 3C

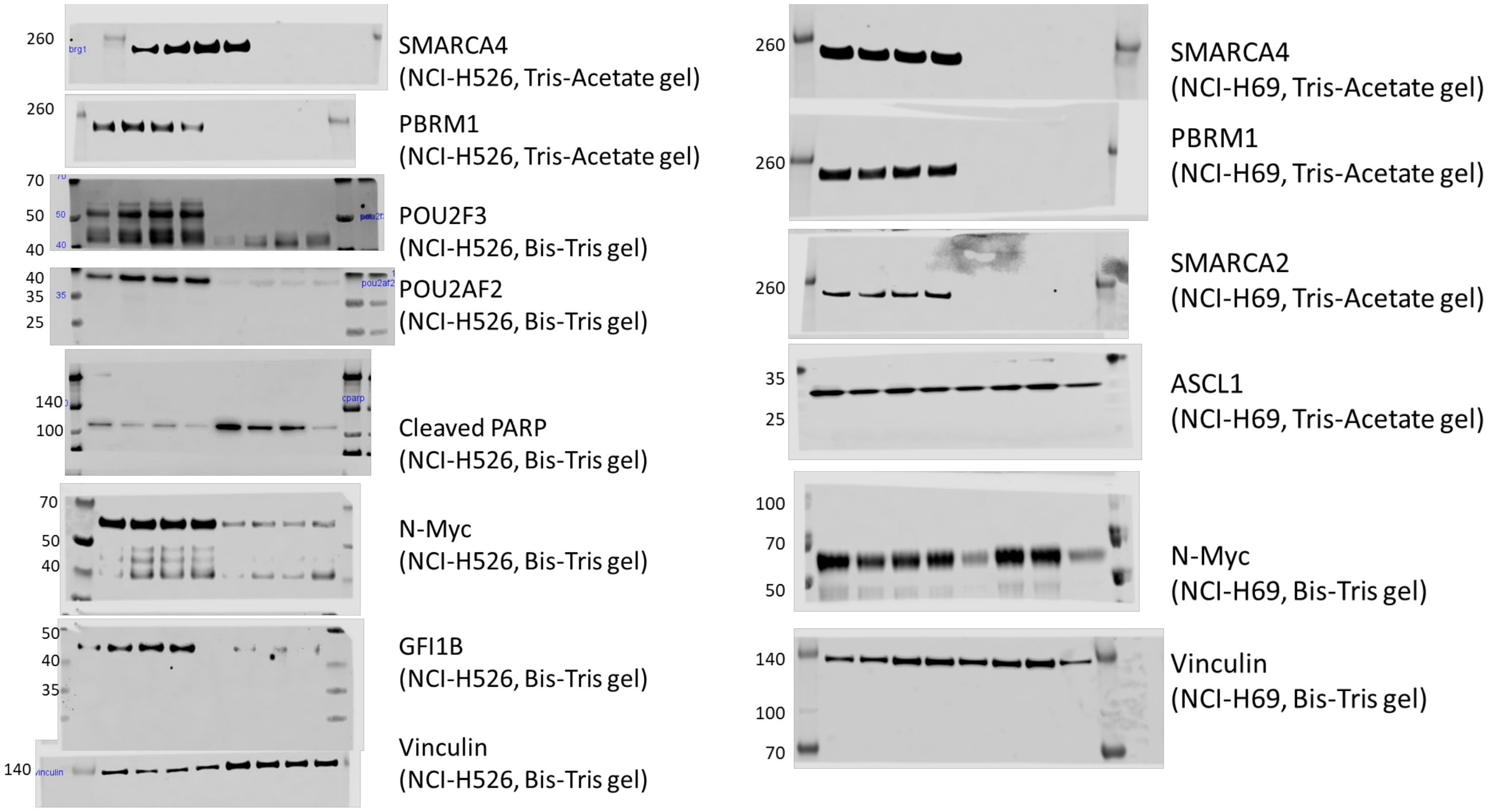
SMARCA4
(NCI-H526, Tris-Acetate gel)
PBRM1
(NCI-H526, Tris-Acetate gel)
POU2F3
(NCI-H526, Bis-Tris gel)
POU2AF2
(NCI-H526, Bis-Tris gel)
Cleaved PARP
(NCI-H526, Bis-Tris gel)
N-Myc
(NCI-H526, Bis-Tris gel)
GFI1B
(NCI-H526, Bis-Tris gel)
Vinculin
(NCI-H526, Bis-Tris gel)
SMARCA4
(NCI-H69, Tris-Acetate gel)
PBRM1
(NCI-H69, Tris-Acetate gel)
SMARCA2
(NCI-H69, Tris-Acetate gel)
ASCL1
(NCI-H69, Tris-Acetate gel)
N-Myc
(NCI-H69, Bis-Tris gel)
Vinculin
(NCI-H69, Bis-Tris gel)
260
260
70
50
40
40
35
25
140
100
70
50
40
50
40
35
140
260
260
260
35
25
100
70
50
140
100
70

Fig S3A

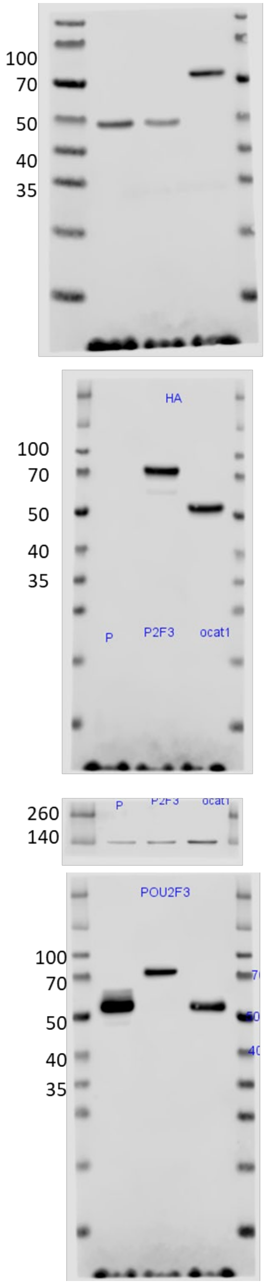

POU2AF2
(NCI-H526, dTAG-HA Bis-Tris gel)

HA-tag
(NCI-H526, dTAG-HA Bis-Tris gel)

Vinculin
(NCI-H526, dTAG-HA Bis-Tris gel)

POU2F3
(NCI-H526, dTAG-HA Bis-Tris gel)

Fig S3B

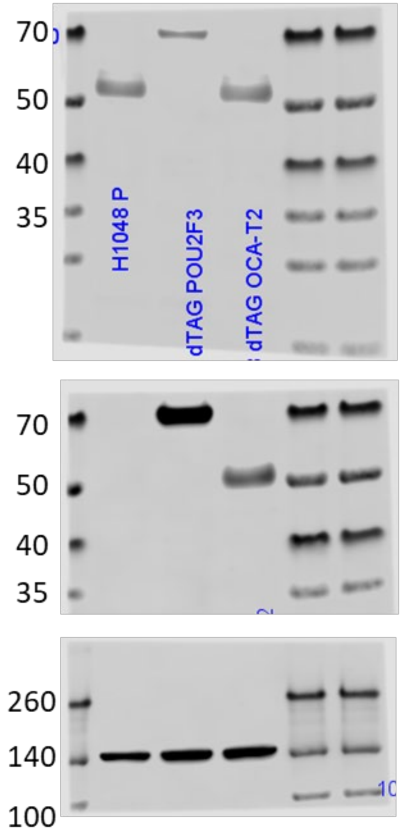

POU2F3
(NCI-H1048, dTAG-HA Bis-Tris gel)

HA-tag
(NCI-H1048, dTAG-HA Bis-Tris gel)

Vinculin
(NCI-H1048, Bis-Tris gel)

Fig S4A (Upper)

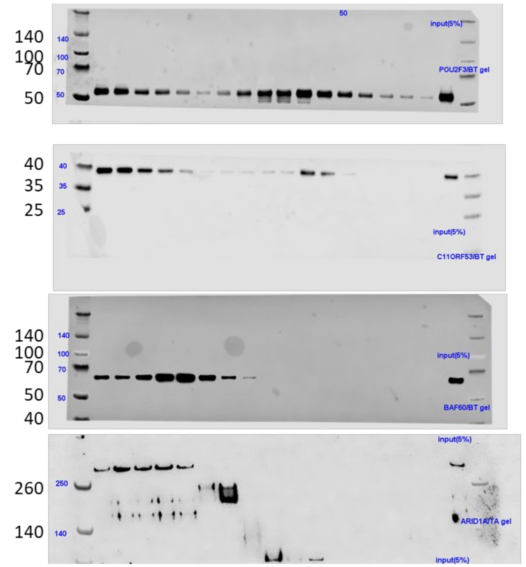

POU2F3 (NCI-H526 Bis-Tris gel)

POU2AF2 (NCI-H526 Bis-Tris gel)

SMARCD1 (BAF60a) (NCI-H526, Bis-Tris gel)

ARID1A (NCI-H526, Bis-Tris gel)

Fig S4A (Bottom)

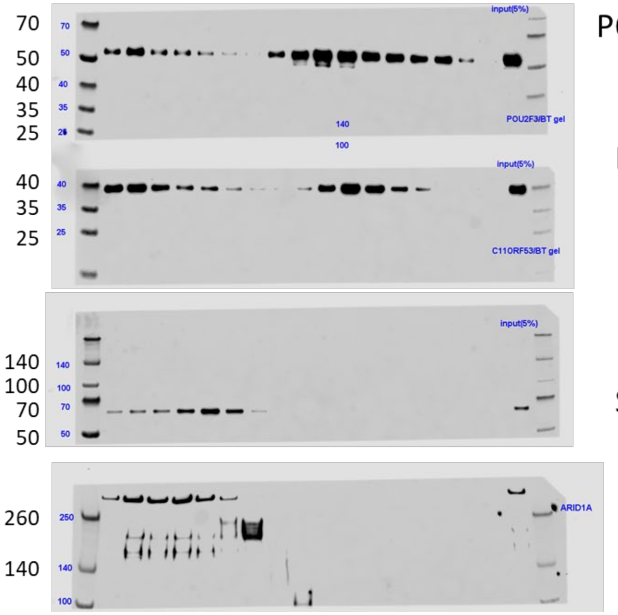

POU2F3 (COR-L311 Bis-Tris gel)

POU2AF2 (COR-L311 Bis-Tris gel)

SMARCD1 (BAF60a) (COR-L311, Bis-Tris gel)

ARID1A (COR-L311, Bis-Tris gel)

Fig S4B

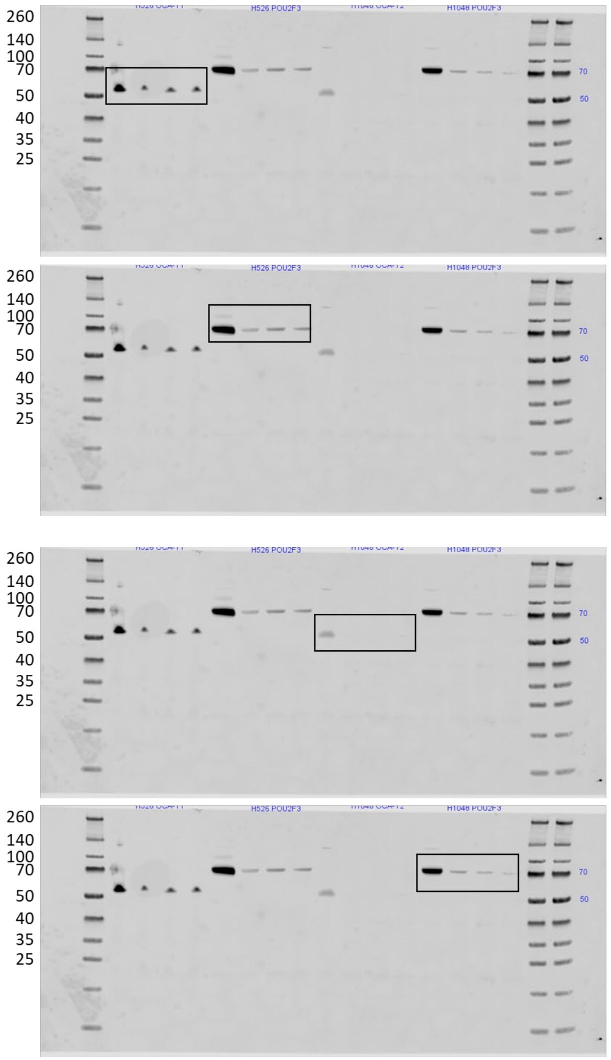

HA-tag (NCI-H526 HA dTAG POU2AF2 Bis-Tris gel)

HA-tag (NCI-H526 HA dTAG POU2F3 Bis-Tris gel)

HA-tag (NCI-H1048 HA dTAG POU2AF3 Bis-Tris gel)

HA-tag (NCI-H1048 HA dTAG POU2F3 Bis-Tris gel)

Fig S7B left

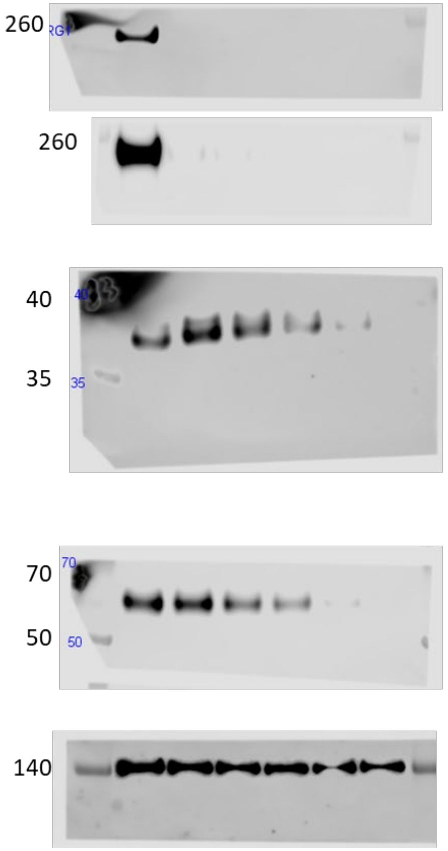

SMARCA4 (MM1.S Tris-Acetate gel)

PBRM1 (MM1.S Tris-Acetate gel)

POU2AF1 (MM1.S, Bis-Tris gel)

C-Myc (MM1.S, Bis-Tris gel)

Vinculin (MM1.S, Bis-Tris gel)

Fig S7B right

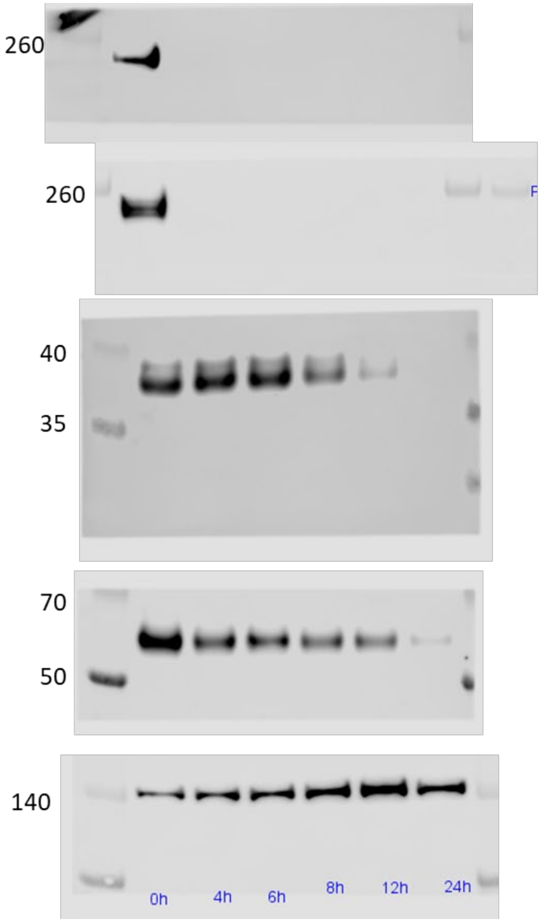

SMARCA4 (NCI-H929 Tris-Acetate gel)

PBRM1 (NCI-H929 Tris-Acetate gel)

POU2AF1 (NCI-H929, Bis-Tris gel)

C-Myc (NCI-H929, Bis-Tris gel)

Vinculin (NCI-H929, Bis-Tris gel)

Fig S9F

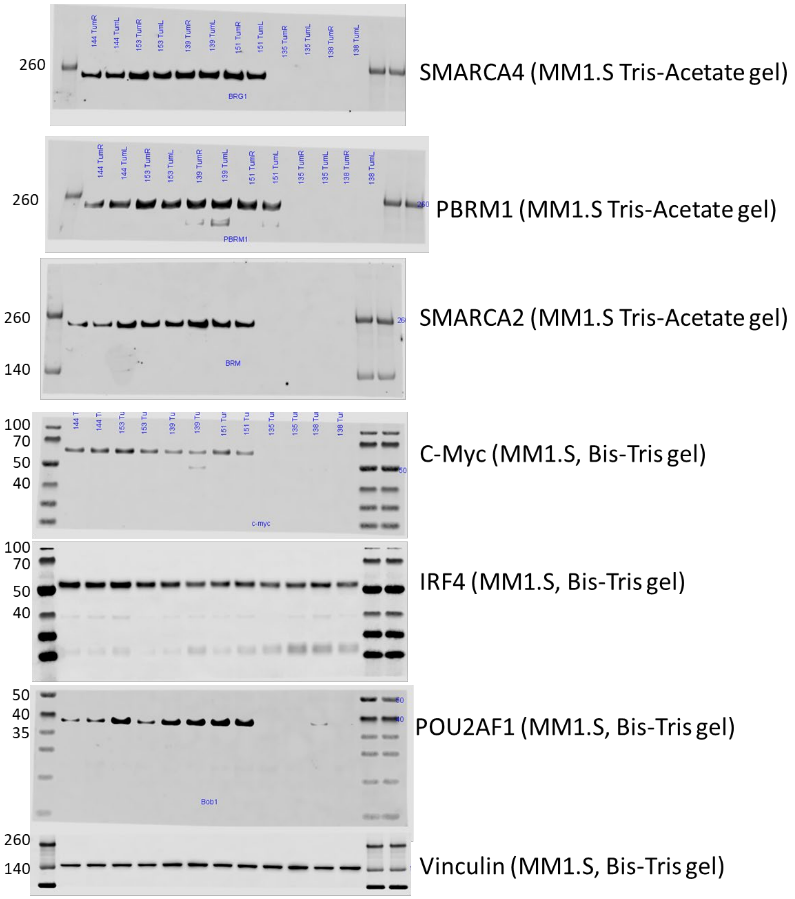
260
SMARCA4 (MM1.S Tris-Acetate gel)
260
PBRM1 (MM1.S Tris-Acetate gel)
260
140
SMARCA2 (MM1.S Tris-Acetate gel)
100
70
50
40
C-Myc (MM1.S, Bis-Tris gel)
100
70
50
40
IRF4 (MM1.S, Bis-Tris gel)
50
40
35
POU2AF1 (MM1.S, Bis-Tris gel)
260
140
Vinculin (MM1.S, Bis-Tris gel)
