# Supplementary material for: Targeting the mSWI/SNF Complex in POU2F-POU2AF Transcription Factor-Driven Malignancies
Source: bioRxiv. 2024 Jan 25:2024.01.22.576669. Preprint. [Version 1] doi: 10.1101/2024.01.22.576669 (PMC10849552; doi:10.1101/2024.01.22.576669)
Supplement: Supplementary file 1 [file NIHPP2024.01.22.576669v1-supplement-4.pdf]

bioRxiv preprint doi: https://doi.org/10.1101/2024.01.22.576669; this version posted January 25, 2024. The copyright holder for this preprint (which was not certified by peer review) is the author/funder, who has granted bioRxiv a license to display the preprint in perpetuity. It is made available under aCC-BY 4.0 International license.

Supplementary Figure Legends

Figure S1. Selective essentiality of the mSWI/SNF complex in POU2F3 molecular subtype of SCLC. Related to Figure 1.
(A) Fold change of positive and negative controls in CRISPR screen.
(B) Beta score for different domains of druggable targeted gene in mSWI/SNF complex in SCLC-P cell lines versus SCLC-A cell lines. RRM, RNA recognition motif.
(C) Immunoblot analysis of indicated proteins in SCLC cells post-treatment with varying time points (right) or concentrations (left two experiments, four hours) of AU-15330. Vinculin serves as the control for protein loading in all immunoblots.
(D) IC_{50} value of five days of AU-15330 treatment for different subtypes of SCLC cell lines.
(E) Representative dose-response curves of SCLC-P and SCLC-A cells treated with AU-15330 at varying concentration for five days.

Figure S2. mSWI/SNF inhibition condenses chromatin at enhancer sites in SCLC cells. Related to Figure 2.
(A) Chromatin compaction induced by mSWI/SNF ATPase degradation. Visualization of ATAC-seq read-density in NCI-H1048 (SCLC-P) post-treatment for 4 hours with either vehicle or 1 μM AU-15330.
(B) Analysis of fold change and significance level for HOMER motifs that are enriched within sites dependent and independent of the mSWI/SNF complex in NCI-H1048 cells.
(C) Genome-wide changes in chromatin accessibility upon AU-15330 treatment for four hours in NCI-H526 (SCLC-P) cells along with genomic annotation of sites that lose physical accessibility (lost) or remain unaltered (retained).
(D) Top five *de novo* motifs (ranked by *p-value*) of mSWI/SNF-dependent sites (top) and mSWI/SNF-independent sites (bottom) enriched within AU-15330-compacted genomic sites (HOMER, hypergeometric test) in NCI-H526 (SCLC-P) cells. POU2F3 (also known as Oct11) is the top motif.
(E) Genome-wide changes in chromatin accessibility upon AU-15330 treatment for 4 hrs in NCI-H1048 (SCLC-P) cells along with genomic annotation of sites that lose physical accessibility (lost) or remain unaltered (retained).
(F) Top five *de novo* motifs (ranked by *p-value*) of mSWI/SNF-dependent sites (top) and mSWI/SNF-independent sites (bottom) enriched within AU-15330-compacted genomic sites (HOMER, hypergeometric test) in NCI-H1048 (SCLC-P) cells. POU2F3 (also known as Oct11) is the top motif.
(G) Genome-wide changes in chromatin accessibility upon AU-15330 treatment for 4 hrs in NCI-H69 (SCLC-A) cells along with genomic annotation of sites that lose physical accessibility (lost) or remain unaltered (retained).
(H) Top five *de novo* motifs (ranked by *p-value*) of mSWI/SNF-dependent sites (top) and mSWI/SNF-independent sites (bottom) enriched within AU-15330-compacted genomic sites (HOMER, hypergeometric test) in NCI-H69 (SCLC-A) cells.

Figure S3. Verification of SCLC POU2F3 and POU2AF2/3 dTAG cell lines. Related to Figure 2.

bioRxiv preprint doi: https://doi.org/10.1101/2024.01.22.576669; this version posted January 25, 2024. The copyright holder for this preprint (which was not certified by peer review) is the author/funder, who has granted bioRxiv a license to display the preprint in perpetuity. It is made available under aCC-BY 4.0 International license.

(A&B) Immunoblots of POU2F3 and HA-tag validate the expression of exogenous fusion dTAG HA-tag POU2F3 and dTAG HA-tag POU2AF2/3 and the knockout efficacy of endogenous POU2F3 and POU2AF2/3 in NCI-H526 (A) and NCI-H1048 (B) dTAG cells. Vinculin is used as a loading control. This experiment was repeated independently twice.
(C) Competition-based proliferation assays in NCI-H211 (left) and NCI-H1048 (right) cells transduced with the indicated sgRNAs with a GFP reporter. The percentage of GFP+ cells correspond to the sgRNA representation within the population. GFP measurements in human cell lines were taken on day 4 post-infection and every four days with Guava Easycyte HT instrument (Millipore). The fold change in GFP+ population (normalized to day 4) was used for analysis.
(D) Relative viability plots after dTAG13 treatment with different time durations.
(E) ChIP–seq read-density heatmaps representing POU2F3 (green), HA-POU2F3 (red), and HA-POU2AF3 (blue) at AU-15330-compacted genomic sites in NCI-H1048 cells following treatment with DMSO or 1 μM AU-15330 for 6 hrs.
(F) ATAC-seq and ChIP-seq tracks for *AVIL*, *PTGS1*, and *ASCL2* in NCI-H1048 with and without 1 μM AU-15330 treatment.

Figure S4. Physical interaction of the POU2F3 complex and the mSWI/SNF complex in SCLC-P cells. Related to Figure 2.
(A) FPLC analysis performed on nuclear extracts of NCI-H526 (SCLC-P) and COR-L311(SCLC-P) cells.
(B) Immunoblot confirming the efficiency of the HA-tag pulldown for HA-POU2F3 and HA-POU2AF2/3 by assessing the presence of residual proteins in the flowthrough samples.
(C-E) TMT-based MS validates the interaction between the mSWI/SNF complex components, POU2F3 and POU2AF2/3 in H526 (C using HA-POU2F3 as bait), H1048 (D using HA-POU2AF3 as bait) and H1048 (E using HA-POU2F3 as bait).
(F) GSEA analysis of 24 hrs of 1 μM AU-15330-induced transcriptomic changes in NCI-H526 and NCI-H1048 cells.

Figure S5. Selective growth inhibition of AU-24118 in SCLC preclinical models. Related to Figure 3.
(A) IC_{50} of AU-24118 in a panel of SCLC cell lines after 5 days of treatment.
(B) Individual tumor weight from vehicle and AU-24118 displayed for NCI-H1048 (left) and NCI-H69 (right). Data are presented as mean+/- SEM. 2-way ANOVA test.
(C) Representative H&E staining with corresponding IHC analyses for SMARCA4, POU2F3, and DCLK1 after 5 days of treatment with AU-24118 in NCI-H1048 xenografts (scale=50μm). The inset scale=20μm.
(D) Representative H&E staining with corresponding IHC analyses for SMARCA4 after 5 days of treatment with AU-24118 in NCI-H69 xenografts (scale=50μm). The inset scale=20μm.

Figure S6. Toxicity evaluation of AU-24118 in SCLC-P xenograft bearing mice. Related to Figure 3.
(A) Percent body weight measurement showing the effect of vehicle and AU-24118 throughout the treatment period for NCI-H526 (left panel), NCI-H1048 (middle panel), and NCI-H69 (right panel) xenografts.

bioRxiv preprint doi: https://doi.org/10.1101/2024.01.22.576669; this version posted January 25, 2024. The copyright holder for this preprint (which was not certified by peer review) is the author/funder, who has granted bioRxiv a license to display the preprint in perpetuity. It is made available under aCC-BY 4.0 International license.

(B) Representative H&E staining with corresponding IHC analyses for SMARCA4 in normal organs (liver, spleen, kidney, small intestine, and lung) (scale=50μm). The inset scale=20μm.

Figure S7. Selective essentiality of the mSWI/SNF complex in B cell malignancies. Related to Figure 4.

(A) Scatter plot depicting gene dependency difference of all B cell malignancies versus other cancer types based on DepMap dataset. The red circles indicate the top 5 essential genes among others.

(B) Immunoblot analysis of indicated proteins in MM1.S and NCI-H929 cells post-treatment with varying time points of 1μM AU-15330. Vinculin serves as the control for protein loading in all immunoblots.

(C) IC_{50} of AU-15330 in a panel of multiple myeloma cell lines after 5 days of treatment.

(D) IC_{50} of AU-15330 in a panel of diffuse large B cell lymphoma cell lines after 5 days of treatment. ABC, activated B cell-like subtype lymphoma; GCB, germinal center B-cell-like subtype lymphoma.

Figure S8. Epigenetic landscape changes in multiple myeloma cells upon treatment with the mSWI/SNF ATPases degrader. Related to Figure 4.

(A) Chromatin compaction induced by mSWI/SNF ATPase degradation. Visualization of ATAC-seq read-density in NCI-H929 cells post-treatment for 4 hours with either vehicle or 1μM AU-15330.

(B&C) Top five *de novo* motifs (ranked by *p-value*) enriched within AU-15330-compacted genomic sites (HOMER, hypergeometric test) in NCI-H929 (B) and MM1.S cells (C).

(D) Genome-wide changes in chromatin accessibility upon AU-15330 treatment for 4 hours in MM1.S cells along with genomic annotation of sites that lose physical accessibility (lost) or remain unaltered (retained).

(E) Genome-wide changes in chromatin accessibility upon AU-15330 treatment for 4 hours in NCI-H929 cells along with genomic annotation of sites that lose physical accessibility (lost) or remain unaltered (retained).

(F) Scatter plot showing the dependency scores for POU2F2/POU2AF1 (left) and IRF4/POU2AF1 (right) in diffused large B cell lymphoma (blue), multiple myeloma (red), and other cancer types based on DepMap dataset.

(G) ChIP–seq read-density heatmaps representing POU2AF1 (blue) and IRF4 (red) at AU-15330-compacted genomic sites in NCI-H929 cells following 6 hrs treatment with DMSO or 1 μM AU-15330.

(H) Top *de novo* motifs (ranked by p-value) enriched within POU2AF1 binding sites (HOMER, hypergeometric test) in MM1.S cells (upper) and NCI-H929 cells (bottom).

(I) Combined ATAC-seq and ChIP-seq tracks for *c-MYC* locus in MM1.S and NCI-H929 cells with and without AU-15330 treatment.

Figure S9. Efficacy of AU-24118 in multiple myeloma subcutaneous xenografts and disseminated tumor model. Related to Figure 4.

(A) Overview of the AU-24118 efficacy study conducted using multiple myeloma subcutaneous xenograft models.

bioRxiv preprint doi: https://doi.org/10.1101/2024.01.22.576669; this version posted January 25, 2024. The copyright holder for this preprint (which was not certified by peer review) is the author/funder, who has granted bioRxiv a license to display the preprint in perpetuity. It is made available under aCC-BY 4.0 International license.

(B) Analysis of tumor volumes in indicated multiple myeloma xenograft models upon treatment with AU-24118 or carfilzomib, measured bi-weekly using calipers (analyzed with a two-way ANOVA test) in the NCI-H929 and Karpas-25 xenografts.
(C) Waterfall plots depicting change in tumor volume at the study endpoint for NCI-H929 and Karpas-25-derived xenograft models.
(D) Individual tumor weights from vehicle, carfilzomib, and AU-24118 treated mice from the NCI-H929 and Karpas-25 xenograft study. Data are presented as mean+/- SEM. 2-way ANOVA test.
(E) Percent body weight measurement showing the effect of vehicle, carfilzomib, pomalidomide, or AU-24118 throughout the treatment period in NCI-H929, Karpas-25, and MM.1S xenograft models.
(F) Immunoblot illustrating levels of the indicated proteins in MM1.S xenografts after AU-24118 treatment for 5 days. Vinculin is utilized as the loading control.
(G) Representative images of GFP/luciferase-expressing MM.1S cells extracted from femur to verify the MM.1S disseminated model.
(H) Quantification of bioluminescence signal indicating the tumor burden (measured once per week using the IVIS Spectrum In Vivo Imaging System) in the MM1.S disseminated xenograft model under different treatments. (Right) Box plot of the bioluminescence quantification at endpoint (day 44) in the MM1.S disseminated xenograft model.
(I) Representative H&E staining with corresponding IHC analyses for SMARCA4 and c-Myc after 5 days of the indicated treatment in MM1.S xenografts (scale=50μm). The inset scale=20μm.
(J) Representative IHC analyses for SMARCA4 and C-Myc after 5 days of the indicated treatment in MM1.S disseminated xenografts (scale=50μm). The inset scale=20μm.

Figure S10. Uncropped and unprocessed immunoblot images. Images are provided for all immunoblots throughout the study, with molecular weight markers indicated.

Supplementary Tables (Excel files)

Supplementary Table 1. Raw data of functional domain-targeted CRISPR/Cas9 screen and data from RIME analysis.

Supplementary Table 2. Antibodies, primers, and other reagents used in the study.
